# Nanocomposite Sprayed Films with Photo-Thermal Properties for Remote Bacteria Eradication

**DOI:** 10.3390/nano10040786

**Published:** 2020-04-20

**Authors:** Mykola Borzenkov, Giuseppe Chirico, Piersandro Pallavicini, Paola Sperandeo, Alessandra Polissi, Giacomo Dacarro, Lavinia Doveri, Maddalena Collini, Laura Sironi, Margaux Bouzin, Laura D’Alfonso

**Affiliations:** 1 Department of Medicine and Surgery, Nanomedicine Center, University of Milano-Bicocca, Via Raoul Follereau 3, 20854 Vedano al Lambro (MB), Italy; 2 Department of Physics, University of Milano-Bicocca, Piazza della Scienza 3, 20126 Milan, Italy; 3 CNR Institute for Applied Science and Intelligent Systems, Via Campi Flegrei 34, 80078 Pozzuoli, Italy; 4 Department of Chemistry, University of Pavia, via Taramelli 12, 27100 Pavia, Italy; 5 Department of Pharmacological and Biomolecular Sciences, University of Milano, via Balzaretti 9, 20133 Milan, Italy

**Keywords:** nanoparticles, photo-thermal effect, nanocomposites, bacteria eradication

## Abstract

Currently there is a strong demand for novel protective materials with efficient antibacterial properties. Nanocomposite materials loaded with photo-thermally active nanoparticles can offer promising opportunities due to the local increase of temperature upon near-infrared (NIR) light exposure capable of eradicating bacteria. In this work, we fabricated antibacterial films obtained by spraying on glass slides aqueous solutions of polymers, containing highly photo-thermally active gold nanostars (GNS) or Prussian Blue (PB) nanoparticles. Under NIR light irradiation with low intensities (0.35 W/cm^2^) these films demonstrated a pronounced photo-thermal effect: Δ*T_max_* up to 26.4 °C for the GNS-containing films and Δ*T_max_* up to 45.8 °C for the PB-containing films. In the latter case, such a local temperature increase demonstrated a remarkable effect on a Gram-negative strain (*P. aeruginosa*) killing (84% of dead bacteria), and a promising effect on a Gram-positive strain (*S. aureus*) eradication (69% of dead bacteria). The fabricated films are promising prototypes for further development of lightweight surfaces with efficient antibacterial action that can be remotely activated on demand.

## 1. Introduction

The limited ability of conventional drugs and agents to eradicate bacteria and biofilms catalyzed the extensive search for new facilities worldwide. Nowadays, a wide range of nanomaterials (both inorganic and organic) demonstrated promising results in antibacterial activity according to numerous studies [1,2,3,4,5,6].

Within the wide variety of existing nanomaterials with antibacterial properties, photo-thermal nanoparticles with absorption in the NIR region are receiving great attention due to the capability to convert absorbed light into heat with subsequent thermal inactivation of different types of bacteria [7,8,9,10]. In addition, NIR light in the so-called biotransparent window (750–900 nm) is considered safe for direct in vivo application as it does not damage normal tissues (at least if used under given irradiance limits, e.g., 0.32 W/cm^2^) and has good penetration depth [11].

Even though different types of photo-thermally active nanoparticles demonstrated efficient antibacterial action under NIR light exposure, there are still issues that limit further implementation of this strategy. Firstly, in the case of nanoparticle colloidal solutions, high laser intensities are usually required to achieve the desired temperatures due to the heat dissipation in the aqueous solvent [7]. Secondly, relatively severe conditions have been used to eradicate bacteria like high laser intensities (>1 W/cm^2^) and high local temperature increases (*T* > 80–200 °C) [12,13,14,15,16,17,18]. Of course, this can be used as a good alternative to conventional sterilization methods but severely limits in vivo application.

The incorporation of photo-thermally active nanoparticles into a polymeric matrix gives instead the opportunity to fabricate uniform materials remotely activated on demand with a high photo-thermal efficiency, preserving the original photo-thermal properties of nanoparticles [19]. This strategy has also promising potential for the preparation of new antibacterial bulk materials for protective wrapping with antibacterial films. For example, chitosan-based hydrogel loaded with gold nanorods showed pronounced antimicrobial activity against both Gram-positive and Gram-negative strains under NIR irradiation [20].

Previously, our group reported fabrication and antibacterial properties of bulk polymeric poly(vinyl alcohol) (PVA) films loaded with gold nanostars (GNS) and Prussian Blue (PB) nanoparticles [21,22] and obtained by solvent casting. The employed GNS displayed two tunable and intense localized surface plasmon resonances (LSPR) in the 700–900 and 1100–1600 nm ranges [7,21]. The local photo-thermal effect triggered by NIR irradiation with 4 W/cm^2^ of films containing GNS (Δ*T* > 100 °C) was highly efficient in killing *E. coli* [21]. Biocompatible and Food and Drug Administration (FDA)-approved PB nanoparticles exhibit intense absorption in the 700–750 nm region due to the metal-to-metal charge transfer between Fe^II^ and Fe^III^ centers of this coordination polymer, and light irradiation of this band results in thermal relaxation [23,24]. NIR irradiation with low intensities (0.3 W/cm^2^) of films loaded with PB nanoparticles resulted in a local increase of temperature (Δ*T* = 78 °C) capable of eradicating *P. aeruginosa* bacteria (76% of dead bacteria) [22].

However, these films suffer from some limitations: they cannot adhere to non planar surfaces and cover large areas and require a relatively large amount of nanoparticles. Spray coating seems to be a promising solution, as this method allows very thin films to be fabricated in a rapid and efficient way with low materials consumption on different bulk surfaces and with various shapes. Film-forming sprays with antibacterial properties containing mupirocin [25], and a supramolecular Fe^III^-tannic acid coordination complex [26] were developed and tested. However, only a few examples of nanoparticles containing sprays for antibacterial coating can be found in the literature. For example, previous publications predominantly focused on silver nanoparticles [27], and ZnO nanoparticles that display well-known intrinsic antibacterial properties [28]. Moreover, antibacterial spray coatings based on photo-thermal nanoparticles are still not reported. Therefore, in this work we introduced spray-coated nanocomposite films and studied their capability to kill bacteria under NIR light irradiation. For this purpose, we developed spray compositions based on poly(vinyl alcohol) and GNS or PB nanoparticles, that can be easily sprayed as films on glass slides. After a detailed study of their photo-thermal properties as a function of laser intensity and laser wavelength, we show that they are capable of eradicating bacteria, both Gram-negative (*P. aeruginosa*) and Gram-positive (*S. aureus*), upon NIR light activation even with low intensities (0.35 W/cm^2^). It is also demonstrated that relatively low local surface temperature increase (<70 °C) is sufficient in almost complete thermal eradication of *P. aeruginosa* and has remarkable effect on *S. aureus* deactivation. These results open new opportunities in the low-cost protective coating of surfaces and instruments with efficient antibacterial action under “mild” conditions and also the possibility to coat implantable medical devices for direct in vivo sterilization, thus avoiding surgical removal in the case of local infection or biofilm formation. 

## 2. Materials and Methods

### 2.1. Materials

Polyethylene glycol *tert*-octylphenyl ether (Triton X-100), chloroauric acid, ascorbic acid, silver nitrate, sodium borohydride were purchased from Sigma-Aldrich (St. Louis, MO, USA) and used as received. Anhydrous citric acid, poly(ethylene glycol) (PEG-200), poly(ethylene glycol) methyl ether thiol (SH-PEG-OCH_3_, Mw 2000 g/mol), iron (III) chloride, potassium hexanocyanoferrate, poly(vinyl alcohol), PVA (Mw 98,000 g/mol), were also commercially available from Sigma-Aldrich (St. Louis, MO, USA) and used as received.

### 2.2. Gold Nanostars (GNS) and Prussian Blue (PB) Nanoparticles Synthesis

GNS were synthesized via seed-growth method and subsequently coated with PEG containing a thiol group according to an established protocol [21,29]. The resulting solution of PEGylated GNS was concentrated ~30 times by means of large volume ultracentrifugation (100 mL) and redissolution of the obtained pellet in a smaller volume (3 mL). PB nanoparticles were prepared according to a previously reported protocol, but increasing from 1 mM to 10 mM the concentrations of the starting Fe^III^ (as FeCl_3_) and Fe^II^ (as K_4_[Fe(CN)_6_]) reagents [22].

### 2.3. General Method of Spray Solutions Containing Nanoparticles Preparation

We dissolved 0.15 g of PVA in 3.5 mL of Milli-Q water and stored 1 h at 80 °C for complete polymer dissolution. We added 0.015 g of PEG-200 and 1.5 mL of PB stock solution or 1.5 mL of PEGylated GNS stock solution successively, and the mixture was stirred 3 h. Then 0.015 g of citric acid was added and the solution was additionally stirred for 1 h at room temperature.

### 2.4. Spray Coating of Glass Disks

Before spray coating glass disks (Zeiss, Oberkochen, Germany, 25 mm of diameter) were washed consistently with ethanol and bi-distilled water and sonicated for 3 min. The bi-distilled water/ultrasound treatment was repeated three times. Then the disks were dried in an oven for 1 h at 100 °C. The spray formulations were placed in the reservoir of an airbrush (Fengda^®^; Zhejiang, China, nozzle diameter 0.3 mm) and sprayed onto glass cover slips using a different number of sprayed layers. The glasses were stored at 130 °C for 1 h to complete cross-linking.

### 2.5. Ultraviolet/Visible/Near-Infrared (UV/VIS/NIR) Spectroscopy

Absorbance spectra of nanoparticles aqueous solutions, spray formulations and resulting sprayed films together with transmittance spectra were recorded using an ultraviolet/visible/near-infrared (UV/VIS/NIR) spectrophotometer V-570 (Jasco, Cremella (LC), Italy).

### 2.6. Contact Angle Measurement

Static contact angle determinations were made with a KSV CAM200 instrument, with the water sessile drop method.

### 2.7. Optical Confocal Microscopy

Raster scanning images of the nanocomposite films were acquired with Leica SP5 TCS confocal microscope (Leica Microsystems, Wetzlar, Germany) in reflection mode with an excitation wavelength of *λ_exc_* = 633 nm and a 20× air immersion objective.

Each image has been acquired with 1024 × 1024 pixels and 400 Hz of line scan frequency, with fields of view ranging from 50 μm × 50 μm to 100 μm × 100 μm. Z-stacks were acquired by taking planes every 0.2 μm. The number of spots in each plane was estimated by employing the Fiji plugin TRackMate [Fiji, version 2.0.0-rc-43/1.52n, NIH] and the intensity distribution of the spots was obtained by the 3D Objects Counter Fiji plugin.

### 2.8. Photo-Thermal Effect of Sprayed Films upon NIR Irradiation

The sprayed films were irradiated with a Mai Tai, NIR laser (Spectra Physics, Santa Clara, CA, USA) with wavelengths tunable in the 700–980 nm range. The diameter of the beam spot was set to 12 mm. The substrate temperature changes were monitored by means of a thermo-vision camera (FLIR, E40, Wilsonville, OR, USA) and the supporting analysis software.

### 2.9. Antibacterial Effect Triggered by NIR Irradiation

*Pseudomonas aeruginosa* PAO1 and *Staphylococcus aureus* ATCC 6538P strains were selected as model Gram-negative and Gram-positive organisms, respectively. Bacteria were routinely grown in LB (Difco, Franklin Lakes, NJ, USA) agar plates, incubating at 37 °C. For antibacterial effect assessment, bacterial cultures were prepared according to the previously reported protocol [22]. Briefly, a single bacterial colony from each strain was inoculated in 5 mL of liquid Mueller–Hinton broth (MH, Difco, Franklin Lakes, NJ, USA) and incubated at 37 °C under shaking for 15 h. Following incubation, bacterial concentration was estimated by spectrometric measurement (V-530, Jasco, Tokyo, Japan) at 600 nm, considering that an OD600 = 1 corresponds approximately to 1 × 10^9^ colony-forming units (CFU)/mL for *P. aeruginosa* PAO1 and to 5 × 10^8^ CFU/mL for *S. aureus* ATCC 6538P. Bacterial cultures were diluted 1:10 with fresh MH broth to adjust the bacterial concentration to approximately 5 × 10^8^ CFU/mL. Glasses with sprayed films were placed in Petri dishes with cover glass bottom (MatTek, Ashland, MA, USA); 20 μL of diluted bacterial suspension was inoculated on the top of the films, and the films were gently air-dried under a laminar flow hood. The films were irradiated with 700 nm (in the case of PB-containing sprayed films) and 850 nm (in the case of GNS-containing sprayed films) laser (0.35 W/cm^2^) and different durations of irradiation. Irradiated and non-irradiated (control) samples were stained with Film Tracer Live/Dead viability kit (L10316, Invitrogen, Carlsbad, CA, USA) based on the use of the SYTO^®^ 9 and propidium iodide stains mixture in an appropriate concentration ratio (0.167 or 3.34:20) to ensure that bacteria with intact membranes (live) stain fluorescent green (SYTO^®^ 9) whereas propidium iodide stains red only bacteria with damaged membranes (dead). The stained samples were analyzed with a Leica SP5 TCS confocal microscope using a 20× dry objective (HC PL FLUOTAR 20 × 0.5, dry, Leica, Wetzlar, Germany). At least three z-stacks of images were collected from three distant regions in the film by using the 488 nm argon ion laser emission in both spectral intervals (510–580 nm and 590–700 nm, where the bleed through of the green dye at the concentration used for the staining experiments is negligible). The images have been analyzed by employing a threshold filter [Fiji, version 2.0.0-rc-43/1.52n, NIH] and measuring the percentual area in the red and green channels [22].

## 3. Results and Discussion

### 3.1. Preparation and Characterization of Sprayed Films Containing GNS and PB Nanoparticles

Two spray formulations were developed as aqueous solution of poly(vinyl alcohol), PVA, (3%, *w*/*v*), with the addition of poly(ethylene glycol), PEG-200, as plasticizer, citric acid as cross-linker (10% with the respect to PVA amount), and PB nanoparticle colloidal solution (30% *v*/*v*) or PEGylated GNS colloidal solution (30% *v*/*v*). The choice of PVA as spray base is justified by its low-toxicity, biocompatibility and excellent film forming properties [21]. The addition of PEG-200 as plasticizer improves the stability of the films [30]. The choice of non-toxic citric acid as cross-linking reagent for PVA was preferred to the toxic glutaraldehyde [31,32].

PB nanoparticles of cubic shape with intense absorption band with *λ_max_* ≈ 700 nm with an average size of 36 ± 10 nm were obtained with ~50% yield. The absorbance spectrum and representative transmission electron microscope (TEM) image of PB nanoparticles are provided in supplementary information, Figure S1.

**Figure 1:**
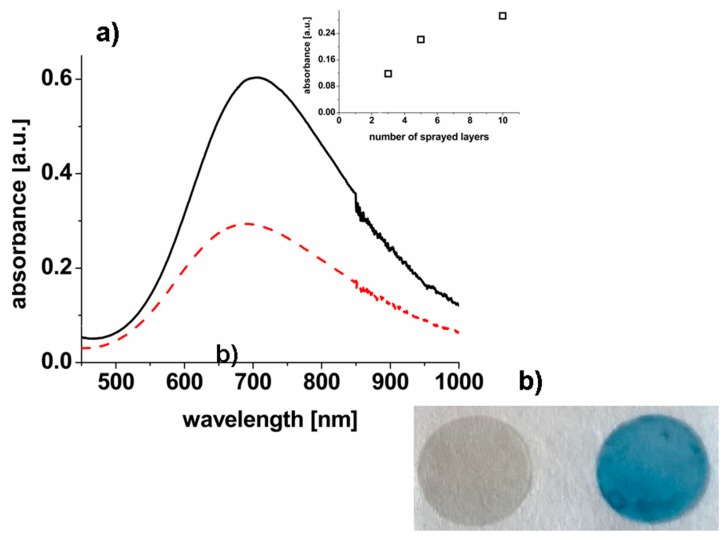
(**a**) Absorbance spectra of Prussian Blue (PB)-based spray formulation (black line; dilution 18 times) and glass with sprayed film (red line, 10 layers). The inset shows the dependence of the absorbance values of sprayed films vs. the number of sprayed layers. (**b**) Pictures of nude glass disk and the glass coated with poly(vinyl alcohol) (PVA)-PB film respectively.

After spray coating, the glasses were stored at 130 °C to complete the cross-linking of PVA films [31]. The absorbance spectra of a spray solution containing PB nanoparticles and a thin film sprayed on glass together with a photo of glasses uncoated and coated with PVA-PB sprayed film are shown in Figure 1.

In order to enhance the photo-thermal effect, the glasses were coated with multiple layers. The absorbance of the sprayed surface increases with the number of layers and saturates at about 10 layers (see inset of Figure 1), that is the number of layers chosen for further experiments. When the cross-linked sprayed film was formed, a slight blue shift of (~16 nm) of NIR absorption maximum was observed (see Figure 1), that may be attributed to the change of local dielectric constant to which charge-transfer bands are sensitive. The PB nanoparticles decreased the transparency of sprayed films: being almost 100% transparent when not loaded with PB nanoparticles they became semi-transparent (e.g., ~56% at 700 nm) in the NIR region (see Figure S2A in supplementary information). The PB nanoparticles remained stable in the spray solution during the storage at ambient conditions and no significant changes in absorbance spectra and nanoparticles size were detected after 4 weeks (see Figures S2B and S3 in supplementary information). The cross-linking of sprayed films is also an essential step for the improvement of their stability. As an example, two glasses coated with cross-linked and non cross-linked sprayed nanocomposite films were immersed in phosphate-buffered saline (PBS) at 37 °C overnight. The non cross-linked film tended to dissolve [30] and a large fraction of the PB nanoparticles was released (data not shown) as can be measured from the increase of the absorbance signal of the buffer at the peak wavelength of the nanoparticles. By contrast, the cross-linked film remained stable and no release of nanoparticles was detected. The contact angle 66.36° ± 0.26° of the film surface indicated hydrophilicity of sprayed films that arise from the hydrophilic nature of PVA and from the surface of PB nanoparticles bearing the citrate capping layer as a stabilizer during their synthesis [32].

**Figure 2:**
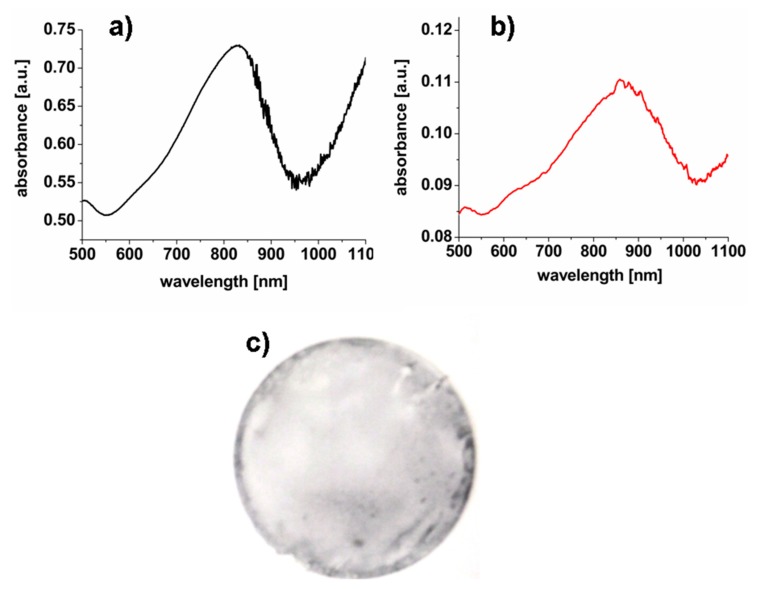
(**a**) Absorbance spectrum of gold nanostars (GNS)-based spray formulation (dilution 5 times). (**b**) Absorbance spectrum of glass with sprayed film containing GNS (10 layers). (**c**) A photo of glass covered with GNS-based film.

GNS were synthesized via a seed-growth technique in the presence of a non-ionic surfactant, Triton X-100, according to a well-established protocol [21]. The as-synthesized GNS are poorly stable when coated only with a layer of surfactant, Triton X-100, which is also known to have some cytotoxicity [21]. Therefore, the GNS were coated with SH-PEG-OCH_3_ (Mw 2000 g/mol) before incorporation into spray formulations. The PEGylated GNS had an average size of 60 ± 10 nm and display two LSPR resonances, of which the first one fell at around 825 nm (see Figure S4 in supplementary information). The GNS spray formulation and the sprayed films (10 layers) were fabricated in the same manner as in the case of PB nanoparticles. The absorbance spectra of a spray solution containing GNS nanoparticles and a thin film sprayed on glass together with a photo of glass coated with PVA-GNS sprayed film are shown in Figure 2.

A slight red shift (~25 nm) of the LSPR absorption maximum was observed (see Figure 2) on the GNS PVA sprayed film. The GNS nanoparticles also decreased the transparency of sprayed films: they became semi-transparent (e.g., ~70.5% at 850 nm) in the NIR region (see Figure S5A in supplementary information). It is notable that GNS in spray formulations were also stable and the absorption peak decreased slightly with the respect to fresh spray solution after 4 weeks of storage at room temperature (see Figure S5B in supplementary information). As in the case of sprayed PB containing films no release of GNS was observed while immersing the glasses in the PBS buffer.

**Figure 3:**
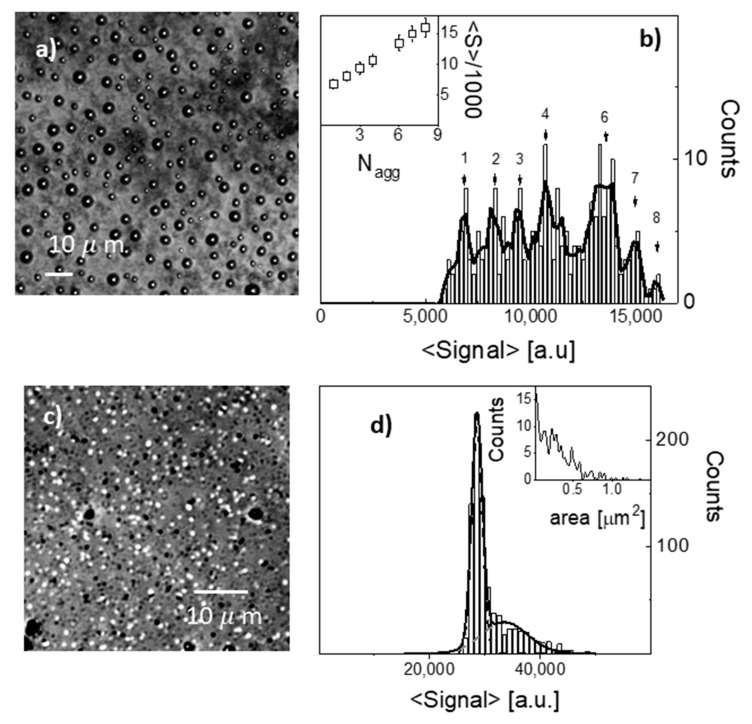
Confocal reflection microscopy characterization. The images are maximum z-projection of a 15-layer stack (200 nm spacing). (**a**) Representative image of PB containing film on glass. (**b**) Distribution of the average signal per spot in the image of panel (**a**). The solid line is the 5-neighbour smoothing of the distribution. The inset reports the average signal of each of the components marked with a vertical arrow in the main panel as a function of a putative aggregation number. (**c**) Representative image of GNS containing film on glass. (**d**) Distribution of the average signal per spot in the image of panel (**c**). The solid line is the best fit to a bimodal Gaussian distribution whose components are reported as dashed lines. The inset reports the distribution of the spot area.

The distribution of the nanoparticles in the sprayed films were characterized by confocal reflection microscopy (see Figure 3a,c). The distributions of the average reflected signal per spot, measured on the nanoparticle-containing films, indicate the presence of aggregates, particularly for the PB case. In this case, the presence in the distribution (Figure 3b) of distinct peaks at equally spaced values of the reflection signal was an indication of small aggregates (up to about 8 nanoparticles, Figure 3b, inset) with similar concentrations. The size of the spots had a unimodal distribution around the spatial resolution size (about 400 × 400 nm^2^). The average concentration of spots was 0.012 +/− 0.002 1/μm^2^. By taking into account the aggregates distribution (Figure 3b) with similar weights on aggregates up to *N_agg_* = 7, we can estimate a PB nanoparticles concentration of about 0.047 +/− 0.006 particles/μm^2^. The aggregation of the PB nanoparticles revealed by Figure 3b could have been induced by multilayer coating followed with cross-linking at 130 °C. For the GNS containing films, the vast majority of the observed spots in the confocal reflection images (Figure 3c) have a well-defined value that corresponds to a dominant component in the signal distribution (Figure 3d) on top of a low and wider component (dashed gray line, Figure 3d) that is an indication of rare, much larger aggregates. The average spot and nanoparticle concentration for the GNS sprayed layers was about 0.14 +/− 0.01 nanoparticles/μm^2^, about 3 times larger than the PB case. It is noteworthy that the moderate aggregation of the nanoparticles did not impact on resulted photo-thermal properties of sprayed films as discussed below. In particular (see Figure 2) in sprayed films GNS plasmon hybridization did not take place. This phenomenon was instead observed when water/alcoholic inks containing PEGylated GNS were printed on glass surfaces, as the PEG coating was not sufficient to keep the inter-particle distance between GNS branches large enough when solvent is removed [33]. In the present paper, we hypothesize that in the dry sprayed films the PVA matrix acts as a barrier, keeping a sufficient inter-particle separation even in the aggregates. 

### 3.2. Photo-Thermal Properties of Sprayed Films Containing PB and GNS Nanoparticles under NIR Light Irradiation

**Figure 4:**
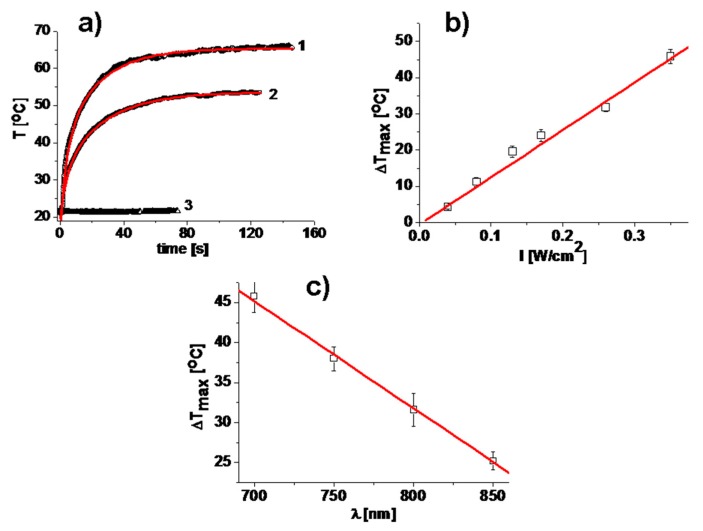
(**a**) Temperature increase of sprayed PB-containing film under irradiation with 700 nm and 0.35 W/cm^2^ (1), 0.26 W/cm^2^ (2); no temperature change is observed for sprayed film without PB nanoparticles, (3) under irradiation with 0.35 W/cm^2^. The data are best fit to double exponential growth (solid red lines). (**b**) Dependence of photo-thermal effect of sprayed PB containing film as a function of irradiation intensity (*λ* = 700 nm). The data are best fit to linear function (red line). (**c**) Dependence of photo-thermal effect of sprayed PB containing film as a function of irradiation wavelength. The data are best fit to linear function (red line).

The photo-thermal properties of sprayed films containing PB nanoparticles were investigated by NIR laser irradiation at *λ* = 700 nm, almost matching with the maximum absorbance of PB nanoparticles in film. In all cases, the temperature increased rapidly, reaching the plateau in less than 20 s as shown in Figure 4a.

The heating kinetics observed on the spray-coated glass slides under NIR irradiation is similar to that found for self-assembled monolayers of PB nanoparticles on glass [32]. The half-maximum raising time was 15 ± 0.19 s and 13.3 ± 0.16 s when the sprayed films were irradiated at 0.26 W/cm^2^ and at 0.35 W/cm. The photo-thermal effect (Δ*T_max_*) reached at these two values of intensities is 30.3° and 45.8°, respectively. As a control, no temperature increase was observed in blank (without PB nanoparticles) PVA sprayed films under NIR irradiation with same intensities, as shown in Figure 4a [21]. As expected, the photo-thermal response increased with increasing irradiation intensity, and it is well described by a linear trend (see Figure 4b). In this study we fixed the maximum intensity of 0.35 W/cm^2^, very close to the maximum allowed exposition of skin (0.32 W/cm^2^) [11]. In addition, we also found that under irradiation with higher intensities, namely 0.51 W/cm^2^ and 0.63 W/cm^2^, the maximum increase of temperature (Δ*T_max_*) was 60 °C and 71 °C, respectively (see Figure S6 in supplementary information). Apart from the laser intensity, the photo-thermal efficiency of the sprayed films depends also on the irradiation wavelength and it decreases when increasing the irradiation wavelength above 700 nm (see Figure 4c). The average ratio of temperatures (Δ*T_max700nm_*/Δ*T_max850nm_* = 1.82) is in agreement with the ratio of the absorbance values of films at corresponding wavelengths (*Abs_700nm_*/*Abs_850nm_* = 1.73). 

The photo-thermal properties of GNS sprayed films were investigated firstly by NIR laser irradiation at *λ* = 800 nm, close to the maximum of the GNS LSPR absorption band in film (Figure 2a).

**Figure 5:**
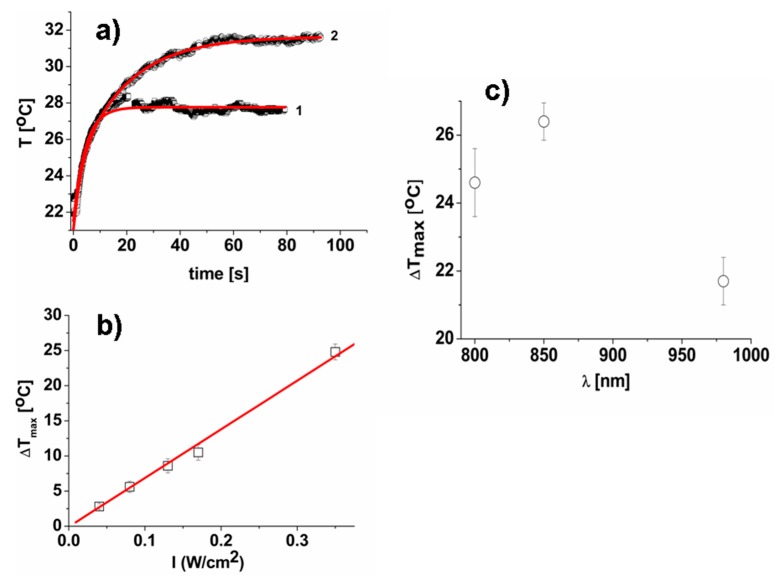
(**a**) The temperature increase of sprayed GNS containing film under irradiation with 800 nm and 0.08 W/cm^2^ (1), 0.17 W/cm^2^ (2). The data are best fit to double exponential growth (solid red lines). (**b**) Dependence of photo-thermal effect of sprayed GNS containing film as a function of irradiation intensity (*λ* = 800 nm). The data are best fit to linear function (red line). (**c**) Dependence of photo-thermal effect of sprayed GNS containing film as a function of irradiation wavelength (laser intensity 0.35 W/cm^2^).

Similar to PB-containing films, also in this case the temperature increased rapidly reaching the plateau in less then 20 s (Figure 5a). The photo-thermal response increased with the increase of irradiation intensity, and it is well described by a linear trend as for the PB-containing films (see Figure 5b). The maximum photo-thermal effect of GNS sprayed films, Δ*T* = 26.4 °C, observed under irradiation with 850 nm laser at 0.35 W/cm^2^ (see Figure 5c), is 2 times lower than the value reached with the PB sprayed films at the same intensity. The average ratio of temperatures (Δ*T_max850nm_*/Δ*T_max800nm_* = 1.06) is in agreement with ratio of absorbance values of GNS-containing films at corresponding wavelengths (*Abs_850nm_*/*Abs_800nm_* = 1.09). As expected, the irradiation of these films at wavelengths different from 850 nm led to a decrease of the photo-thermal effect of sprayed films. For example, Δ*T_max_* 21.7 °C, was observed under irradiation with a 980 nm laser at 0.35 W/cm^2^ (see Figure 5c). Also in this case the average ratio of temperatures (Δ*T_max850nm_*/Δ*T_max980nm_* = 1.22) is in agreement with the ratio of absorbance values of GNS-containing films at corresponding wavelengths (*Abs_850nm_*/*Abs_980nm_* = 1.21).

It is noteworthy that the photo-thermal effect from the sprayed films is very similar to that obtained from hydrogels containing the same type of nanoparticles [22], in spite of the fact that in hydrogels a much larger amount of nanoparticles have been used. In fact, starting from PVA/nanoparticle solutions with the same nanoparticle concentration, a total volume of 5 mL over a diameter of 5 cm was used for the hydrogels preparation, while in this work c.a. 100 μL of solutions were sprayed overall (on 10 layers) to cover an area of diameter 2.5 cm. However, taking into account the almost two orders of magnitude difference in the thickness of the hydrogel films and the sprayed layers (the thickness estimated from confocal reflection microscopy is about 100 micrometers and 1 micrometer for the two cases, respectively), we can estimate a very similar concentration of the nanoparticles in the two cases. Still, taking into account the shined volume, the amount of nanoparticles shined by the NIR beam on the hydrogel films is about one order of magnitude larger than that on the 10 sprayed layers. The comparable photo-thermal effect measured in the two cases stems, then, from two major reasons. The nanoparticles are all lying on the same plane in the sprayed films with minimal possibility of mutual shadowing of the NIR radiation. Moreover, the amount of water in the sprayed layers is minimal and the nanoparticles are dissipating heat mostly in air when on the sprayed layers, compared to the case of PVA hydrogels loaded with nanoparticles. The final result is that the sprayed films are much more efficient in terms of photo-thermal effect per nanoparticles unit. 

### 3.3. Photo-Thermally Induced Bacteria Eradication of Sprayed Films Containing PB and GNS Nanoparticles Triggered by NIR Light

**Figure 6:**
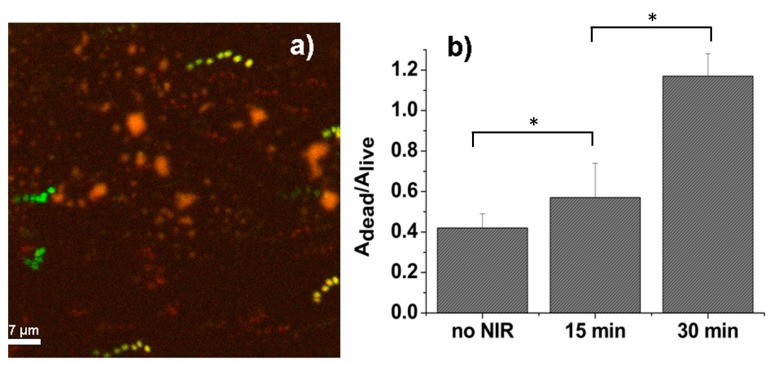
(**a**) The representative confocal image of *P. aeruginosa* bacteria on sprayed film after 15 min of NIR irradiation. Field of view: 64.2 × 64.2 μm^2^; (**b**) the dependence of ratio A_dead_/A_live_ as a function of NIR exposure duration on *P. aeruginosa* inoculated on GNS-containing films. The error bars correspond to the standard deviation on samples of at least 70 bacteria per image. An asterisk indicates that the two-samples *t*-test *p* < 0.001.

First, we studied the antibacterial properties of sprayed film containing GNS nanoparticles under NIR light exposure. For this purpose *P. aeruginosa* cultures were inoculated onto the films that were then irradiated with NIR light (850 nm; 0.35 W/cm^2^, Δ*T_max_* 26.4 °C) for 15 and 30 min. *P. aeruginosa* is known to be one of the most common opportunistic human pathogens and with high adaptability to environmental changes [34,35]. Therefore, it is essential to develop new and lightweight approaches aimed at its efficient eradication. After irradiation, the samples were stained and analyzed by means of confocal microscopy and compared to control (not irradiated) samples. After segmentation of the red emitting (propidium iodide stained, dead) cells and the green emitting (SYTO^®^ 9 stained, alive) cells, the antibacterial effect was measured as the ratio of the red to the green area of the image, A_dead_/A_live_ [22]. The results of the photo-thermally induced antibacterial effect of sprayed GNS containing films and representative confocal image are shown in Figure 6.

It is noteworthy that we found also that non-irradiated sprayed films demonstrated slight antibacterial effect (A_dead_/A_live_ = 0.42 ± 0.11, corresponding to 30% of dead cells). Such results are not related to the presence of gold nanoparticles, as gold is not intrinsically antibacterial [21]. As a matter of fact, also the sprayed films without GNS nanoparticles demonstrated a comparable antibacterial effect (A_dead_/A_live_ = 0.5 ± 0.5, corresponding to 34% of dead bacteria after 3 h of inoculation). These values are slightly higher than the intrinsic mortality of bacteria seeded on inert substrates (A_dead_/A_live_ = 0.33 ± 0.06 on sterilized glass slides 3 h after inoculation, corresponding to 25% of dead bacteria). NIR irradiation during 15 min of the GNS films slightly increased the antibacterial effect (A_dead_/A_live_ = 0.57 ± 0.07, corresponding to 37% of dead cells). However, even increasing the NIR duration to 30 min did not lead to a remarkable antibacterial effect (A_dead_/A_live_ = 1.17 ± 0.11, corresponding to 54% of dead cells). This can be explained by fact that the maximum increase of temperature of GNS-containing films was lower than 50 °C under tested irradiation conditions (see Figure 5). Previously it was reported that enzymes, proteins, and lipids in bacteria would become denatured and metabolism would be affected when the temperatures rise above 50 °C [36]. 

**Figure 7:**
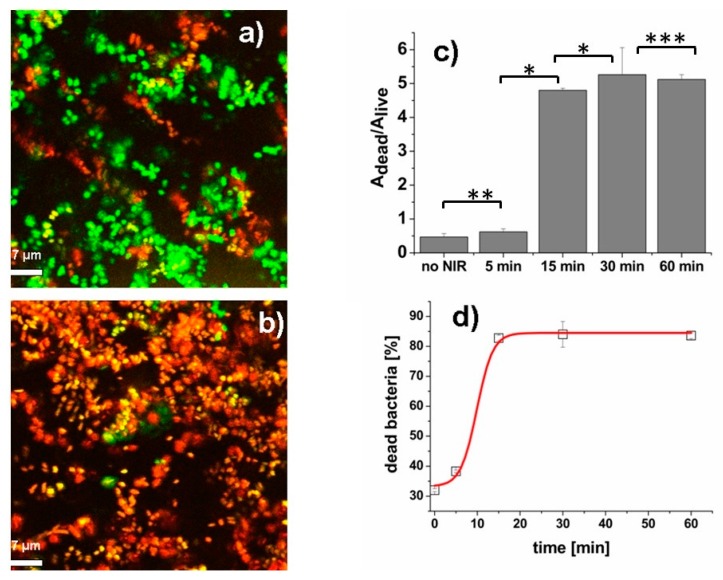
(**a**) The representative confocal image of *P. aeruginosa* bacteria on sprayed PB-containing film without NIR irradiation. Field of view: 64.2 × 64.2 μm^2^; (**b**) the representative confocal image of bacteria on sprayed film after 15 min of NIR irradiation. Field of view: 64.2 × 64.2 μm^2^; (**c**) The dependence of ratio A_dead_/A_live_ as a function of NIR exposure duration; (**d**) The dependence of dead bacteria fraction (%) as a function of NIR irradiation duration. The data are best fit to sigmoidal function (red line). The error bars of the data are the standard deviations computed on a sample of at least 70 bacteria per image. The symbols “*”, “**” and “***” indicates that the two-samples *t*-Test provides *p* < 0.001, *p* = 0.009 and *p* = 0.43, respectively.

As PB-containing sprayed films demonstrated more pronounced photo-thermal effect under same laser intensity (Δ*T* = 45.8 °C), their antibacterial properties were studied in detail. The representative confocal images and results of photo-thermally induced antibacterial effect of PB-containing sprayed films against *P. aeruginosa* are shown in Figure 7.

Also in this case, non-irradiated sprayed films demonstrated slight antibacterial effect (A_dead_/A_live_ = 0.47 ± 0.1, corresponding to 32% of dead cells). Such results are not connected to the presence of PB nanoparticles, as they are not able to statically release bactericidal metal ions [32]. The short term (5 min) NIR irradiation of the PB-containing films increased the fraction of dead bacteria with respect to the control (A_dead_/A_live_ = 0.62 ± 0.09, corresponding to 38.3% of dead bacteria). The photo-thermal induced antibacterial effect on *P. aeruginosa* cells of the sprayed films increasing with increasing the irradiation duration: we measured A_dead_/A_live_ = 4.8 ± 0.06 and A_dead_/A_live_ = 5.26 ± 1.1 for irradiation times of 15 min and 30 min, respectively (see Figure 7c). These values correspond to 82.75% of dead bacteria in the case of 15 min irradiation and 84% in the case of 30 min irradiation, respectively (see Figure 7d). No significant increase of the bacterial death was found for 60 min NIR irradiation (see Figure 7c,d). Our results indicate, therefore, the promising opportunity to eradicate bacteria under significantly lower temperatures than those reported in the literature. Sprayed films capable of eradicating bacteria at such low temperatures can open wide possibilities of their application on soft materials that cannot stand higher temperatures.

**Figure 8:**
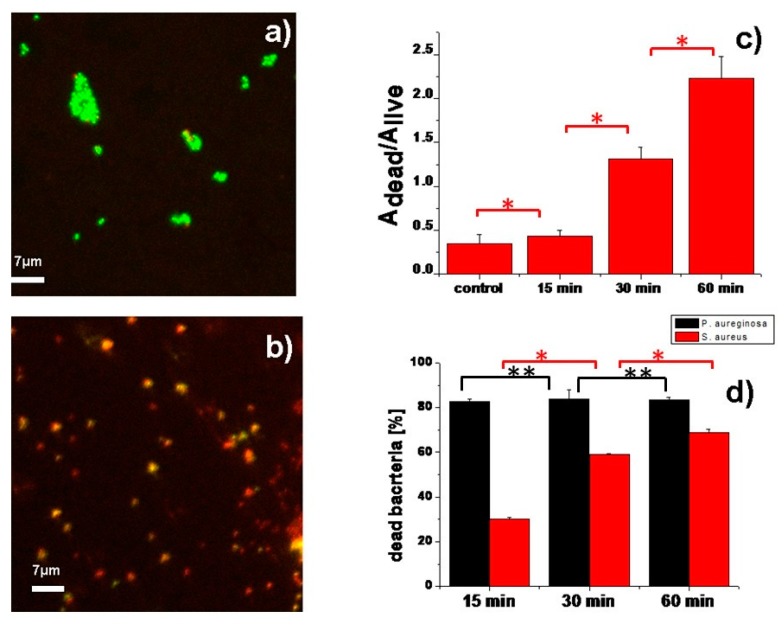
(**a**) The representative confocal image of *S. aureus* bacteria on PB-containing sprayed film without NIR irradiation. Field of view: 64.2 × 64.2 μm^2^; (**b**) presentative confocal image of S. aureus bacteria on PB-containing sprayed film after 30 min of NIR irradiation. Field of view: 64.2 × 64.2 μm^2^; (**c**) The dependence of ratio Adead/Alive of *S. aureus* bacteria as a function of NIR duration (the symbol “*” indicates that the two-samples *t*-test *p* < 0.001); (**d**) comparison of fraction of dead *P. auereginosa* and *S. aureus* bacteria upon NIR irradiation under same conditions. The error bars correspond to the standard deviation on samples of at least 70 bacteria per image. The symbol “*” indicates that the two-samples *t*-test *p* < 0.001. The symbol “**” indicates that *p* > 0.43.

We also studied the possibility to induce the death of Gram-positive bacterial cells seeded on PB-containing films, under the same irradiation conditions. *S. aureus* is a major Gram-positive human pathogen that causes a wide range of clinical infections [37]. The samples preparation and analytical technique were identical as in the previous case (see Experimental details for detailed information). *S. aureus* inoculated films were irradiated with NIR light (700 nm; 0.35 W/cm^2^) for 15, 30 and 60 min and the results are shown in Figure 8 together with summarized impact of NIR-induced photo-thermal effect on both bacteria strains.

Also in this case, non irradiated control films displayed slight antibacterial effect (A_dead_/A_live_ = 0.35 ± 0.1 corresponding to 26% of dead bacteria). Again, these values were slightly higher than the intrinsic mortality of *S. aureus* bacteria inoculated onto inert substrates (A_dead_/A_live_ = 0.23 ± 0.04 on sterilized glass slides, corresponding to 19% of dead bacteria). The representative confocal images of non irradiated and irradiated cells are shown in Figure 8a,b. After 15 min of NIR irradiation of the sprayed film, the fraction of dead bacteria increased to A_dead_/A_live_ = 0.43 ± 0.07, corresponding to 30% of dead bacteria (see Figure 8c). However, the antibacterial efficiency against *S. aureus* was lower than that measured for *P. aeruginosa*, as can be expected from the known higher resistance of Gram-positive bacteria to heat, due to their thicker cell wall with respect to the Gram-negative ones [38]. The eradication of *S. aureus* cells increased steadily under irradiation: A_dead_/A_live_ = 1.31 ± 0.14 for 30 min irradiation time and A_dead_/A_live_ = 2.23 ± 0.25 for 60 min irradiation time (see Figure 8c), even though we could not reach the same values as observed for the Gram-negative strain. These A_dead_/A_live_ ratios correspond to 59% of dead bacteria in case of 30 min irradiation and 69% in case of 60 min irradiation respectively (see Figure 8d).

Finally, we studied the possibility to increase the fraction of dead *S. aureus* bacteria under irradiation with higher laser intensity. After the 30 min NIR irradiation at 0.63 W/cm^2^ the ratio of A_dead_/A_live_ increased to 2.8 ± 0.15, corresponding to 73.7% of dead bacteria (see representative confocal image in Figure S7 in supplementary information).

## 4. Conclusions

Hybrid materials based on nanoparticles can offer new and efficient methods of antibacterial treatment. Among existing types of nanoparticles, photo-thermally active nanomaterials are particularly promising due to their capacity for the thermal eradication of bacteria upon NIR light exposure. However, in order to enhance the impact of these nanoparticles in the practical field and foster industrialization, we need to tackle issues related to reduce the laser intensities needed to reach temperature sufficient to eradicate and find the possibility to cover large surfaces with low materials consumption. As a possible solution, we introduced spray formulation containing highly photo-thermally active nanoparticles capable of forming protective thin films. The sprayed formulation displayed long-term stability during storage at ambient conditions. Moreover, the films obtained by multiple layers deposition demonstrated high photo-thermal efficiency upon NIR light activation at laser intensities within the safety levels for medical applications. It is particularly noteworthy that the material consumption in producing the sprayed layers is about 2% in comparison with nanocomposite hydrogels. We also demonstrated that GNS containing sprayed layers have only a moderate antibacterial effect against *P. auereginosa* strains (54% of dead bacteria), while PB containing films were highly efficient in eradicating *P. aeruginosa* (up to 84% of dead bacteria) and *S. aureus* cells (up to 69% of dead bacteria). 

In our opinion, the possibility to skip the high-temperature cross-linking and to avoid the usage of any toxic reagents in the formulation of the composites to be sprayed will be a further future major improvement in our formulation. However, the results presented here already promote the development of remotely activated antibacterial coatings based on the photo-thermal treatment of the surface at temperatures (65–67 °C) that are lower than those typically employed in other thermal sterilization techniques and can also be directly applied for treatment of everyday uses equipment, of medical devices and prostheses before and after the implantation. 

## Supplementary Materials

The following are available online at https://www.mdpi.com/2079-4991/10/4/786/s1: Figure S1: A: absorbance spectrum of PB stock aqueous solution (diluted 36 times); B: representative TEM image of PB nanoparticles.; Figure S2: A: The transmittance spectra of sprayed films (10 layers) with (blue line) and without (black line) Prussian Blue nanoparticles; B: Absorbance spectra of PB spray formulation immediately after preparation (black line) and after 4 weeks of storage (red line); Figure S3: Size distribution of PB nanoparticles in spray solution after 4 weeks of storage; Figure S4: Absorbance spectrum of GNS stock aqueous solution (diluted 7 times); Figure S5: A: The transmittance spectra of sprayed (10 layers) GNS-PVA film; B: Absorbance spectra of GNS spray formulation immediately after preparation (black line) and after 4 weeks of storage (red line); Figure S6: The temperature increase of sprayed film under irradiation with 700 nm and laser intensity of 0.63 W/cm^2^ (1) and 0.51 W/cm^2^ (2). The data are best fit to double exponential growth (solid red lines); Figure S7: Representative confocal image of *S. aureus* bacteria after 30 min of NIR irradiation at 0.63 W/cm^2^. Field of view: 64.2 × 64.2 μm^2^.
