# Supplementary material for: Nanocomposite Sprayed Films with Photo-Thermal Properties for Remote Bacteria Eradication
Source: Nanomaterials (Basel). 2020 Apr 20;10(4):786. doi: 10.3390/nano10040786 (PMC7221876; doi:10.3390/nano10040786)
Supplement: Supplementary file 1 [file nanomaterials-10-00786-s001.pdf]

SUPPLEMENTARY INFORMATION

Nanocomposite Sprayed Films with Photo-Thermal Properties for Remote Bacteria Eradication

Mykola Borzenkov [1,*], Giuseppe Chirico [2,3], Piersandro Pallavicini [4], Paola Sperandeo [5], Alessandra Polissi [5], Giacomo Dacarro [4], Lavinia Doveri [4], Maddalena Collini [2,3], Laura Sironi [2], Margaux Bouzin [2] and Laura D'Alfonso [2,*]

1 Department of Medicine and Surgery, Nanomedicine Center, University of Milano-Bicocca, Via Raoul Follereau 3, 20854, Vedano al Lambro (MB), Italy;
2 Department of Physics, University of Milano-Bicocca, Piazza della Scienza 3, 20126 Milan, Italy; giberto.chirico@mib.infn.it (G.C.); maddalena.collini@mib.infn.it (M.C.); laura.sironi@unimib.it (L.S.); margaux.bouzin@unimib.it. (M.B.)
3 CNR Institute for Applied Science and Intelligent Systems, Via Campi Flegrei 34, 80078, Pozzuoli, Italy;
4 Department of Chemistry, University of Pavia, via Taramelli 12, 27100 Pavia, Italy; psp@unipv.it (P.P.); giacomo.dacarro@unipv.it (G.D.); laviniarita.doveri01@universitadipavia.it (L.D.)
5 Department of Pharmacological and Biomolecular Sciences, University of Milano, via Balzaretti 9, 20133 Milan, Italy; paola.sperandeo@unimi.it (P.S.); alessandra.polissi@unimi.it (A.P.)
* Correspondence: mykola.borzenkov@unimib.it (M.B.); laura.dalfonso@unimib.it (L.D.)

Received: 24 March 2020; Accepted: 16 April 2020; Published: 20 April 2020

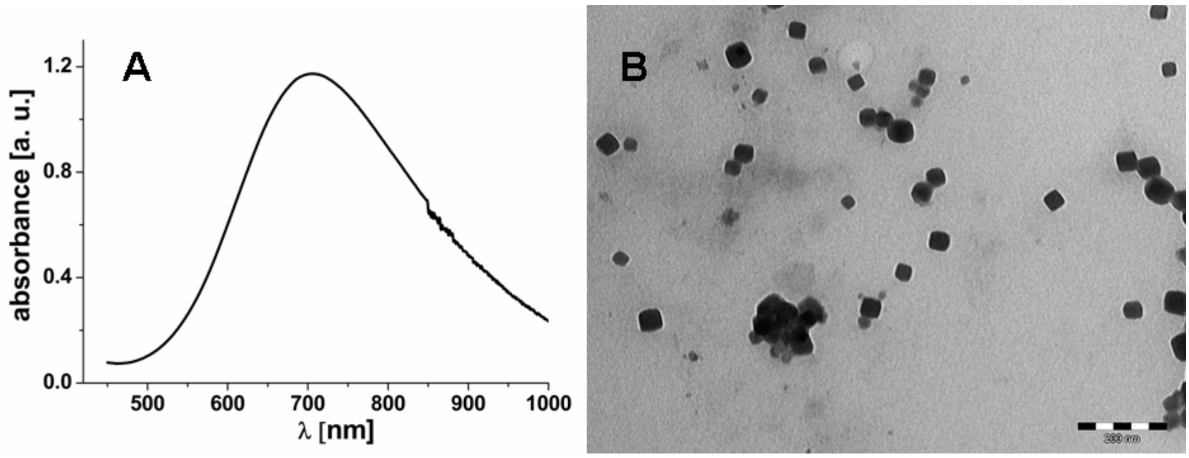

Figure S1. **A**: absorbance spectrum of PB stock aqueous solution (diluted 36 times); **B**: representative TEM image of PB nanoparticles.

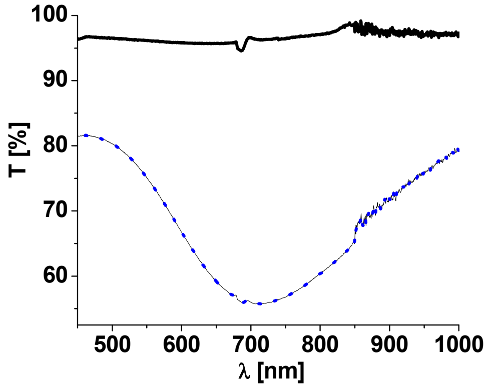

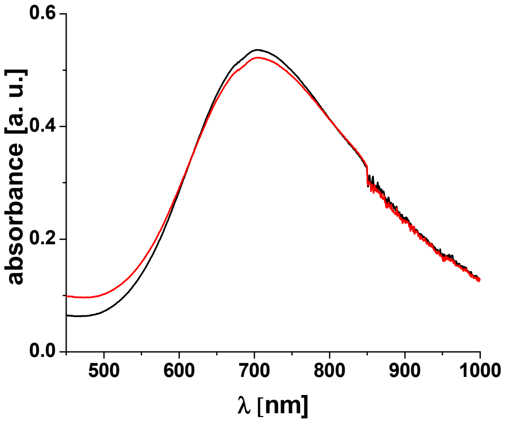

Figure S2A. The transmittance spectra of sprayed films (10 layers) with (blue line) and without (black line) Prussian Blue nanoparticles

Figure S2B. Absorbance spectra of PB spray formulation immediately after preparation (black line) and after 4 weeks of storage (red line)

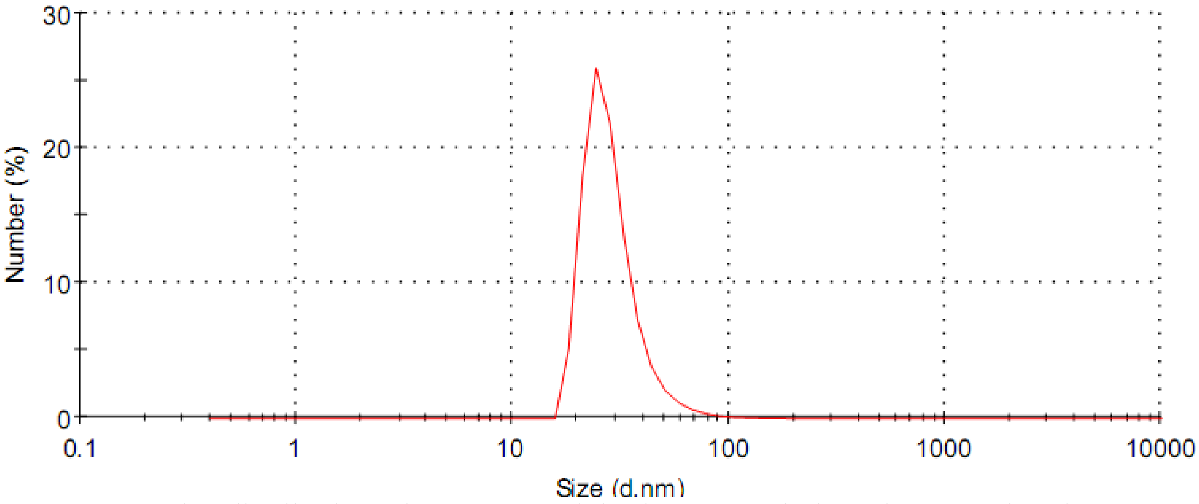

Figure S3. Size distribution of PB nanoparticles in spray solution after 4 weeks of storage

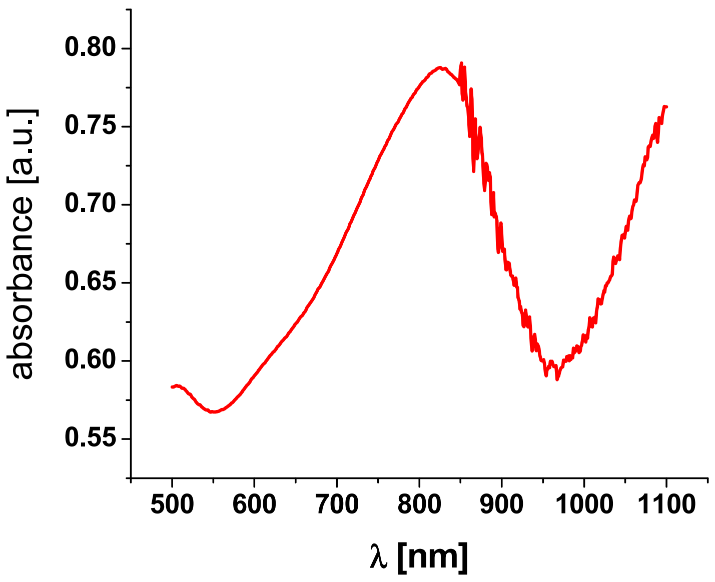

Figure S4. Absorbance spectrum of GNS stock aqueous solution (diluted 7 times)

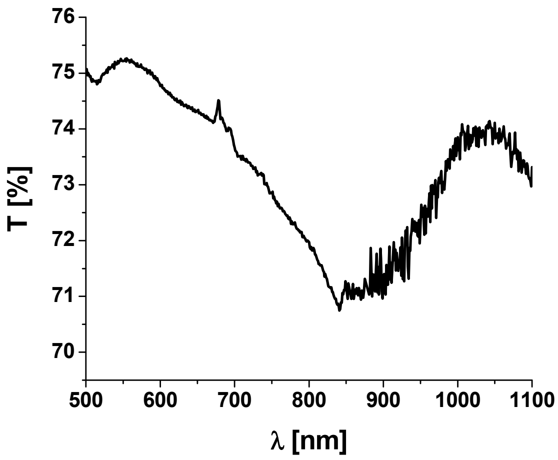

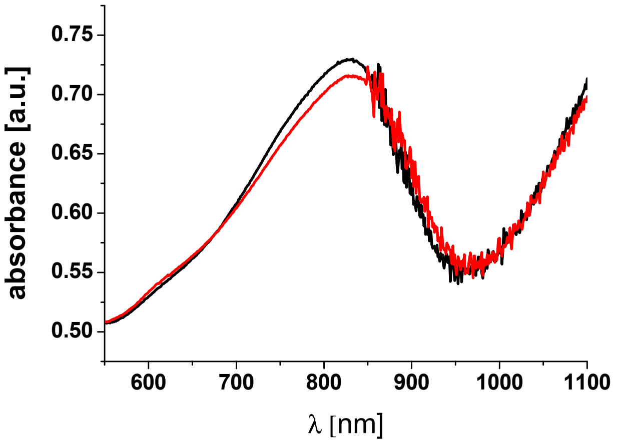

Figure S5A. The transmittance spectra of sprayed (10 layers) GNS-PVA film

Figure S5B. Absorbance spectra of GNS spray formulation immediately after preparation (black line) and after 4 weeks of storage (red line)

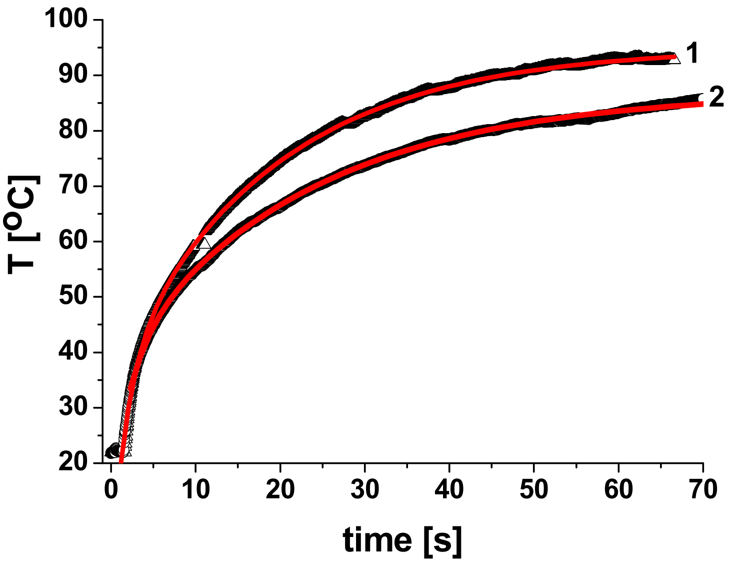

Figure S6. The temperature increase of sprayed film under irradiation with 700 nm and laser intensity of 0.63 W/cm^2 (1) and 0.51 W/cm^2 (2). The data are best fit to double exponential growth (solid red lines).

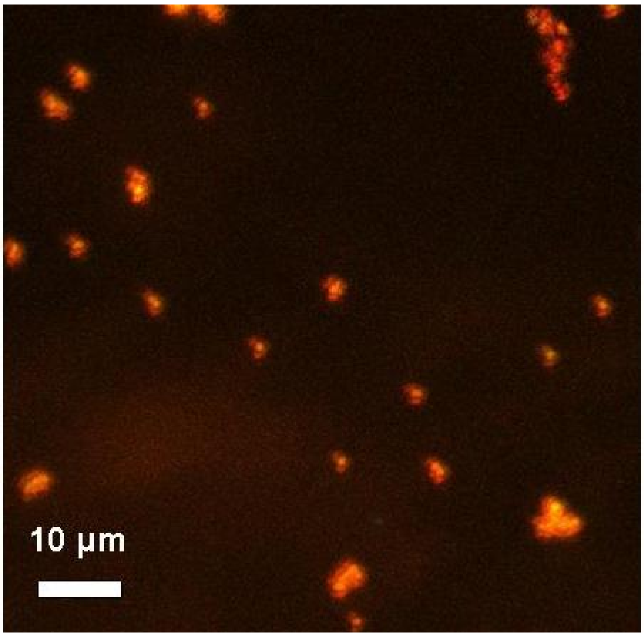

Figure S7. Representative confocal image of *S. aureus* bacteria after 30 min of NIR irradiation at 0.63 W/cm^2. Field of view: 64.2x 64.2 μm^2.
